# Cardiomyopathy Associated with Celiac Disease in Childhood

**DOI:** 10.1155/2012/170760

**Published:** 2012-10-10

**Authors:** Aleksandra Boskovic, Ivana Kitic, Dragan Prokic, Ivica Stankovic

**Affiliations:** Department of Gastroenterology and Hepatology, Mother and Child Health Care Institute, Radoja Dakica 4-6, Belgrade 11070, Serbia

## Abstract

Celiac disease is predominantly a disease of the small intestine characterized by chronic malabsorption in genetically susceptible individuals who ingest grains containing gluten, such as wheat, barley, and rye. Although previously believed to be uncommon, celiac disease may be present in up to 1% of the adult and children population. Celiac disease is associated frequently with iron-deficiency anemia, dermatitis herpetiformis, selective IgA deficiency, thyroid disorders, diabetes mellitus, and various connective tissue disorders but is rarely associated with cardiomyopathy.

## 1. Introduction

Celiac disease is characterized by chronic malabsorption in susceptible individuals who ingest grains containing gluten, such as wheat, barley, and rye. Until recently, celiac disease was believed to be relatively uncommon. However, a recent report from Finland estimates its prevalence at 1% in the general population [1]. Celiac disease is associated frequently with iron-deficiency anemia, dermatitis herpetiformis, selective IgA deficiency [2], thyroid disorders [3], diabetes mellitus [4, 5], and various connective tissue disorders [6–9]. Cardiomyopathy associated with celiac disease is reported infrequently [10–13].

## 2. Report of a Case

A 3-years- old girl, with family antecedents of a sister who died at 9 months and a brother who died at 6 months in circumstances which are not determined, was admitted to our department with a dyspnea and life-threatening cardiomiopatia dilatativa.

Results of a physical examination revealed the following: failure to thrive, a blood pressure of 87/48 mm Hg, a heart rate of 176 beats/min with galloping rhythm, and respiratory rate 57 resp/min with pulmonary crackles in bases. Findings were normal for the rest of the physical examination.

**Figure 1:**
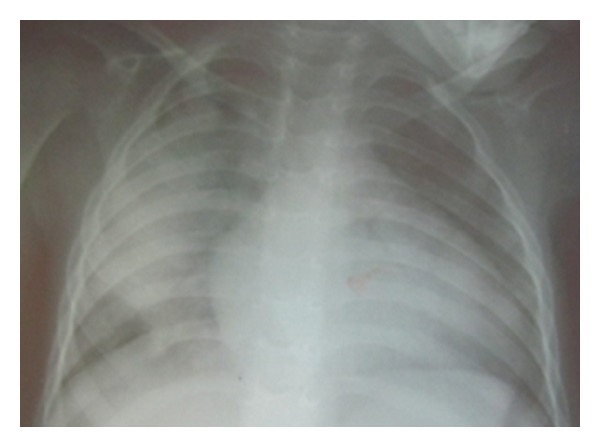
The thorax X-ray shows a cardiomegaly.

 Laboratory findings show a hypochrome microcytic anemia (Hg 6.6 gr/dL), hypoalbuminaemia (27 g/L), hypertransaminasemia (ALT 448, AST 114 ij/L), high creatine phosphokinase (CPK) level (1280 ij/L) with MB fraction 76 ij/L (reference range 19 ij/L). Cholesterol, triglyceride levels, and renal function tests were within normal limits. Chest radiography revealed cardiomegaly and right pulmonary consolidation, and echocardiography showed global hypokinesis with a left ventricular dilatation EDD 41 mm and ejection fraction of 39–45%, without valvular problem and an elevated pressure filling of the left heart (Figure 1). The pulmonary arterial pressures were also elevated. Serology testings for influenza A, influenza B, adenovirus, RSV, and parainfluenza were negative.

Tissue transglutaminase antibody level was positive >200 U/mL (reference range, <10 U/mL) and anti-endomysial IgA antibody titre was positive (1 : 160). She was typed for HLADQ B1∗02, ∗03 DRB1∗03∗04. by polymerase chain reaction.

**Figure 2:**
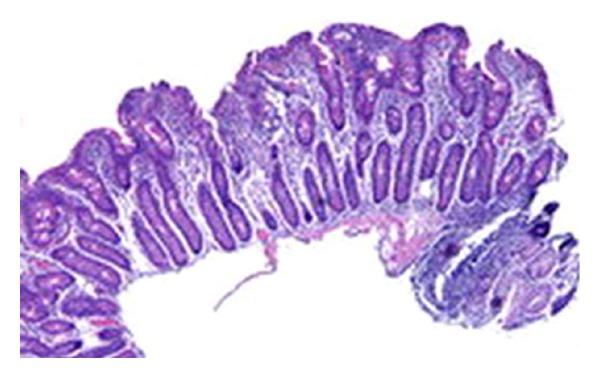
Villous atrophy grade 3c according to Marsh's classification.

**Figure 3:**
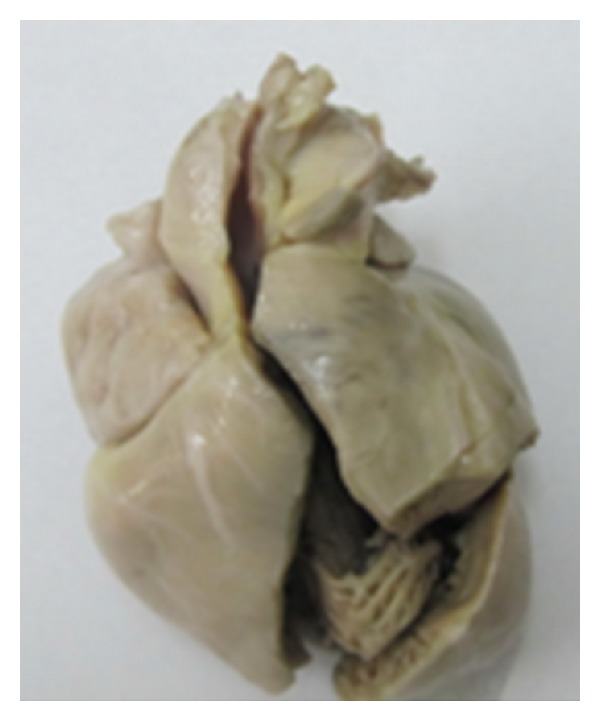
Macroscopic view of the heart at autopsy.

On the third day of hospitalisation the patient died. The anatomopathologic exam of duodenum in autopsy shows the aspect of a villous atrophy stage Marsh 3c thus confirming the diagnosis (Figure 2). The anatomopathologic exam of heart in autopsy shows dilated cardiomyopathy (Figure 3). The diagnosis of dilated cardiomyopathy (DMC) associated to celiac disease has been kept.

The retrospective review of the medical file of her sister shows that she was taken care for celiac disease discovered in front of a microcytary hypochrome anemia at 7.5 gr/dL of hemoglobin which was explored.

## 3. Discussion

Celiac disease is predominantly a disease of the small intestine that develops in genetically susceptible individuals after dietary exposure to grains containing gluten. Ingestion of gluten results in inflammation of the intestinal mucosa along with hyperplasia of the crypts and atrophy of the villi of the small intestine. The inflammatory response is believed to be mediated by immune mechanisms. Classical findings of CD usually begin at 1–3 years of life. Toddlers and young children classically present with chronic diarrhea, vomiting, poor appetite, abdominal distension, abdominal pain, irritability, and failure to thrive after the introduction of gluten in the diet [6]. Other manifestations include osteomalacia, coagulopathy, and peripheral neuropathy. A gluten-free diet usually results in complete resolution of the symptoms and correction of the metabolic abnormalities.

Dilated cardiomyopathy is the most commonly seen type of cardiomyopathy. Regarding its etiology, genetic causes, endocrine disorders, collagen vascular diseases, drugs, congenital metabolism diseases, muscular dystrophies, structural heart diseases, acute and chronic myocarditis, and toxins can be present. However, 50% of cases remain idiopathic. An increased incidence of CD in patients with idiopathic dilated cardiomyopathy as well as in patients with secondary cardiomyopathy has been reported recently [10]. In our case, dilated cardiomyopathy was diagnosed with echocardiography.

Cardiomyopathy associated with celiac disease is infrequent. A study of 52 patients reported an incidence of celiac disease (5.8%) in patients with dilated cardiomyopathy [11]. In another study of 275 patients with heart failure, the incidence of celiac disease was 1.9% versus 0.35% in the control group [12]. Frustaci et al. reported a greater than 4% incidence of celiac disease in 277 consecutive patients with myocarditis [10]. In a study of 60 people with celiac disease aged greater than 65 years, 5% died secondary to heart failure, significantly higher than in the nonceliac disease population [10]. Barrio et al. presented a first case of a severe progressive dilated cardiomyopathy that required heart transplantation in young woman with celiac disease [14].

Regarding pediatric population there are only few cases of celiac disease and associated myocarditis or cardiomyopathy reported [15, 16]. Frustaci et al. reported few cases of lymphocytic myocarditis and celiac disease in childhood [10]. Doğan et al. reported one 8-year-old girl with stroke and dilated cardiomyopathy [15]. Lakhhdar et al. reported the case of two brothers (25 and 23 years) and their sister (who died at 14) presenting a DCM and for whom the celiac disease was discovered whereas they presented no digestive symptoms [17]. The discovery of celiac disease in subjects presenting a DCM of nondetermined etiology raises the question of its possible relation of cause and effect in particular when the situation occurs in the same family [17].

Several mechanisms have been proposed for the development of cardiomyopathy in celiac disease. Nutritional deficiencies secondary to chronic malabsorption may lead to cardiomyopathy. A study in patients with cardiomyopathy associated with celiac disease showed a greater decrease in serum total carnitine levels than in patients with isolated cardiomyopathy [18, 19]. Celiac disease causes increased systemic absorption of various luminal antigens and infectious agents, which may cause myocardial damage secondary to immune-mediated mechanisms [20]. Myocardial injury can also be attributed to immune response cross-reactivity against antigens present in the small intestine and the myocardium [10, 12, 16]. In a case study of cardiomyopathy with celiac disease, similar cellular changes were found in intestinal microvilli and cardiac muscle [21]. However, a study to investigate celiac disease in patients with cardiomyopathy and their relatives concluded that celiac disease seems to be associated, but not cosegregated, with dilated cardiomyopathy in familial cases [22]. Interestingly, celiac disease is associated with a decrease in ischemic heart disease [23]. Compared to a normal population, patients with celiac disease have lower cholesterol, lower triglycerides, lower apolipoprotein B, lower fibrinogen, and higher HDL [24].

Our patient was typed for HLA DQ B1∗02,∗03 DRB1∗03∗04. In some reports we found that in DMC there is a greater incidence of B15 (20 versus 6%) and DQ3 (83 versus 50%) antigens [25].

Our case highlights several important points about celiac disease and associated conditions. Celiac disease in childhood can be asymptomatic or presents with extremely few symptoms. Cardiomyopathy associated with celiac disease is a serious and potentially lethal condition. It is very rare especially in early childhood, but still present. All the pediatric patients who present with DMC or myocarditis in the absence of known etiologies should be carfully investigated. The discovery of celiac disease associated with a DMC of nondetermined etiology raises the question of mechanism of this association in the goal of new therapeutic option.

## References

[1] Mäki M, Mustalahti K, Kokkonen J (2003). Prevalence of celiac disease among children in Finland. *The New England Journal of Medicine*.

[2] Meini A, Pillan NM, Villanacci V, Monafo V, Ugazio AG, Plebani A (1996). Prevalence and diagnosis of celiac disease in IgA-deficient children. *Annals of Allergy, Asthma and Immunology*.

[3] Counsell CE, Taha A, Ruddell WSJ (1994). Coeliac disease and autoimmune thyroid disease. *Gut*.

[4] Cronin CC, Feighery A, Ferriss JB, Liddy C, Shanahan F, Feighery C (1997). High prevalence of celiac disease among patients with insulin-dependent (type I) diabetes mellitus. *The American Journal of Gastroenterology*.

[5] Talal AH, Murray JA, Goeken JA (1997). Celiac disease in an adult population with insulin-dependent diabetes mellitus: use of endomysial antibody testing. *The American Journal of Gastroenterology*.

[6] Farrell RJ, Kelly CP (2002). Celiac sprue. *The New England Journal of Medicine*.

[7] Rustgi AK, Peppercorn MA (1988). Gluten-sensitive enteropathy and systemic lupus erythematosus. *Archives of Internal Medicine*.

[8] Komatireddy GR, Marshall JB, Aqel R, Spollen LE, Sharp GC (1995). Association of systemic lupus erythematosus and gluten enteropathy. *Southern Medical Journal*.

[9] Collin P, Reunala T, Pukkala E, Laippala P, Keyrilainen O, Pasternack A (1994). Coeliac disease—associated disorders and survival. *Gut*.

[10] Frustaci A, Cuoco L, Chimenti C (2002). Celiac disease associated with autoimmune myocarditis. *Circulation*.

[11] Curione M, Barbato M, De Biase L, Viola F, Lo Russo L, Cardi E (1999). Prevalence of coeliac disease in idiopathic dilated cardiomyopathy. *The Lancet*.

[12] Prati D, Bardella MT, Peracchi M, Porretti L, Scalamogna M, Conte D (2002). Antiendomysial antibodies in patients with end-stage heart failure. *The American Journal of Gastroenterology*.

[13] Gasbarrini G, Ciccocioppo R, De Vitis I, Corazza GR, Club del Tenue Study Group (2001). Coeliac disease in the elderly. A multicentre Italian study. *Gerontology*.

[14] Barrio JP, Cura G, Ramallo G (2011). Heart transplantation in rapidly progressive end-stage heart failure associated with celiac disease. *BMJ Case Reports*.

[15] Doğan M, Peker E, Cagan E (2010). Stroke and dilated cardiomyopathy associated with celiac disease. *World Journal of Gastroenterology*.

[16] Curione M, Barbato M, Viola F, Francia P, De Biase L, Cucchiara S (2002). Idiopathic dilated cardiomyopathy associated with coeliac disease: the effect of a gluten-free diet on cardiac performance. *Digestive and Liver Disease*.

[17] Lakhhdar R, Ben Slima H, Drissa M, Drissa H (2012). Family dilated cardiomyopathy associated to celiac disease. *La Tunisie Medicale*.

[18] Curione M, Danese C, Viola F (2005). Carnitine deficiency in patients with coeliac disease and idiopathic dilated cardiomyopathy. *Nutrition, Metabolism and Cardiovascular Diseases*.

[19] Uslu N, Demir H, Karagöz T, Saltik-Temizel IN (2010). Dilated cardiomyopathy in celiac disease: role of carnitine deficiency. *Acta Gastro-Enterologica Belgica*.

[20] van Elburg RM, Uil JJ, Mulder CJJ, Heymans HSA (1993). Intestinal permeability in patients with coeliac disease and relatives of patients with coeliac disease. *Gut*.

[21] Chuaqui B, Garrido J, Casanegra P (1986). Actin-deficient cardiomyopathy coexisting with celiac disease:a chance association?. *Pathology Research and Practice*.

[22] Not T, Faleschini E, Tommasini A (2003). Celiac disease in patients with sporadic and inherited cardiomyopathies and in their relatives. *European Heart Journal*.

[23] Whorwell PJ, Alderson MR, Foster KJ (1976). Death from ischaemic heart-disease and malignancy in adult patients with coeliac disease. *The Lancet*.

[24] Lear JT, Neary RH, Jones P, Fitzgerald DA, English JSC (1997). Risk factors for ischaemic heart disease in patients with dermatitis herpetiformis. *Journal of the Royal Society of Medicine*.

[25] Osa A, Almenar L, Palencia M, Puig N (1999). Antigens of the major histocompatibility system in ischemic heart disease and idiopathic dilated cardiomyopathy. *Clinical Cardiology*.

